# Postbiotics Enhance NK Cell Activation in Stress-Induced Mice through Gut Microbiome Regulation

**DOI:** 10.4014/jmb.2111.11027

**Published:** 2022-03-09

**Authors:** Ye-Jin Jung, Hyun-Seok Kim, Gunn Jaygal, Hye-Rin Cho, Kyung bae Lee, In-bong Song, Jong-Hoon Kim, Mi-Sun Kwak, Kyung-Ho Han, Min-Jung Bae, Moon-Hee Sung

**Affiliations:** 1 Department of R&D Research Center, KookminBio Corporation, Seoul 02826, Republic of Korea; 2 Department of Bio and Fermentation Convergence Technology, Kookmin University, Seoul 02707, Republic of Korea; 3 Technical Assistance Department (R&D Department), The Food Industrial Promotional Agency of Korea, Iksan 54576, Republic of Korea; 4 Osstem Implant Co., Ltd., Bio R&D Center, Seoul 07789, Republic of Korea

**Keywords:** Postbiotics mixture, natural killer cell, fecal microbiome, immune response

## Abstract

Recent studies have revealed that probiotics and their metabolites are present under various conditions; however, the role of probiotic metabolites (*i.e.*, postbiotics in pathological states) is controversial. Natural killer (NK) cells play a key role in innate and adaptive immunity. In this study, we examined NK cell activation influenced by a postbiotics mixture in response to gut microbiome modulation in stress-induced mice. In vivo activation of NK cells increased in the postbiotics mixture treatment group in accordance with Th1/Th2 expression level. Meanwhile, the Red Ginseng treatment group, a reference group, showed very little expression of NK cell activation. Moreover, the postbiotics mixture treatment group in particular changed the gut microbiome composition. Although the exact role of the postbiotics mixture in regulating the immune system of stress-induced mice remains unclear, the postbiotics mixture-induced NK cell activation might have affected gut microbiome modulation.

## Introduction

Interest in probiotics-based functional foods has increased considerably in recent years, largely due to their enormous potential for improving health [1]. Among the most common probiotics are *Bifidobacterium* and *Lactobacillus*. Their actions in humans and in animal models reveal distinct cellular and molecular mechanisms [2-4]. The immunomodulation effects and production of antimicrobial molecules play major roles in the positive efficacy of oral probiotics, and contribute to modulating the immune status of the host [5].

In addition, several recent studies have examined the positive effect of probiotic supplementation on the activation of natural killer (NK) cells as innate immunity [6]. Such actions also influence immunosenescence, highlighting the fact that commensal intestinal bacteria effectuate immune development and function [7-10]. Enhanced NK cell function by probiotics supplementation has been proven in a previous meta study of healthy older people [11]. Moreover, probiotics in the intestine modulate microbiota function, directly triggering an immune reaction from M cells, macrophages, or dendritic cells. However, their role in the immune response is still unclear [12]. Nevertheless, these positive probiotic functions have been clearly demonstrated. The metabolites of *Lactobacillus* spp., *Bifidobacterium* spp., and *Bacillus* spp., in particular, directly affect antivirulence activity [13]. Furthermore, mechanism studies revealed this to be an important element of probiotic bacteria [14].

Probiotic metabolites have also been called cell-free supernatants (CFS) or postbiotics [15], while postbiotics were recently and tentatively defined as health-promoting bioactive compounds produced during the fermentation process (including inactive microbial cells, cell constituents, and metabolites). It was also reported that postbiotics inhibit cytokine-induced apoptosis and reduce the impact of therapeutic metabolic disease [16-20]. As such, there is now a growing interest in ameliorating inflammation related to immune response. For example, the alteration of a macrophage phenotype and attenuation of cytokine expression by probiotics and postbiotics have been demonstrated [21-27]. Although the exact mechanism of their action is still not fully understood, postbiotics have been shown to possess a direct therapeutic mechanism regarding the human intestinal aspect of macrophages and immune status [28].

In this regard, our aim in this study was to explore whether postbiotics mixtures affect the immune status in response to immune suppression in stress-induced mice.

## Materials and Methods

### Composition of Postbiotics Mixture

Three bacterial species from among the three probiotic bacterial strains that showed resistance to low pH and bile salts were selected from Korean traditional foods. There were two strains of *Lactobacillus plantarum* [KM1, KM2], and one strain of *Bacillus velezensis* [KMU01]. These strains were identified using 16S rRNA gene sequence analysis. *Lactobacillus* and *Bacillus* were 1% (v/v) inoculated into MRS and TSA broth media, respectively, and cultured at 37°C for 24 h until the number of cells reached 10^9^ CFU/ml. The probiotics culture supernatant was collected by centrifugation at 10,000 ×*g* for 30 min at 4°C. Each separated culture supernatant was filtered with a 0.22 um syringe. Finally, the probiotics cell-free supernatant was mixed in the following ratio and used as a research sample. (*L. plantarum* KM1: *L. plantarum* KM2: *B. velezensis* KMU01 = 1:1:1).

### Analysis of Free Amino Acids

The free amino acid concentration of postbiotics mixtures was determined using a Biochrom 30+ high-performance liquid chromatography cation exchange system (Biochrom, UK) capable of detecting ninhydrin derivatives. A sample was centrifuged for 5 min in a high-speed centrifuge to remove the precipitate and obtain the supernatant. Finally, after filtration through a 0.2 μm filter, the supernatant was injected into the instrument. The ion exchange column separated the free amino acids of the sample, and the amino acid analyzer performed a derivatization reaction with ninhydrin for detection at 570 nm. Peaks of samples were identified and quantified by comparison with a known standard. Standards for analysis were calibrated using basic amino acid standards (A6282, Sigma-Aldrich, USA) and acidic/neutral amino acid standards (A6407, Sigma-Aldrich).

### Cell Culture and Treatment

The RAW264.7 cell line was purchased from the Korean Cell Line Bank (40071; Korea) and cultured in Dulbeccós modified Eaglés medium (DMEM; SH30243.01, Hyclone, USA) supplemented with 10% fetal bovine serum (FBS; #16000-036, Gibco, USA), 100 U/ml penicillin, 100 μg/ml streptomycin, and Fungizone^®^ (amphotericin B) 0.25 μg/ml (Antibiotic-Antimycotic; #15240-096, Gibco) at 37°C in a 5% CO_2_ atmosphere with media changes every 48 h. To determine that the immune-related cytokine was TNF-α, cells were seeded at a density of 2 × 10^5^ cells/well in 24-well plates in standard growth medium. The next day, the cells were treated with lipopolysaccharide (LPS; L6529, Sigma-Aldrich) and Probiotics Culture Media Supernatant (*L. plantarum* KM1, *L. plantarum* KM2, *B. velezensis* KMU01 postbiotics mixtures = 1:1:1, volume). Then, the TNF-α expression was measured using an ELISA analysis kit (#552268, BD, USA).

### Animals and Experimental Design

Male six-week-old C57BL/6N mice were obtained from Samtaco Co. (Korea). Mice were acclimated under a 12 h light/dark cycle at 23 ± 2°C for 2 weeks in a specific pathogen-free animal facility at FOODPOLIS, Korea National Food Clusters (Korea). They were fed a standard diet and given water ad libitum. All animal experiment protocols were approved by the Institutional Animal Care and Use Committee of FOODPOLIS, Korea National Food Clusters (IACUC-20-006(**A**)).

We used the mouse forced swimming test to evaluate the effect of the Probiotics Culture Media Supernatant (*L. plantarum* KM1: *L. plantarum* KM2: *B. velezensis* KMU01 = 1:1:1) on the immune status according to a previously reported method [29]. After acclimation, the animals were randomized and assigned to one of four groups before the study began [30]. Group (1), the native group of mice received saline by daily gavage and was not forced to do the swimming test. Group (2) was forced to swim, but as the control (CON) group, these mice received only saline by daily gavage. Group (3) was forced to swim and received 400 μl of postbiotics mixtures by daily gavage. Group (4) was forced to swim and received 300 mg/kg of Red Ginseng (RG) by daily gavage [31]. Three groups were forced to swim because this is known to suppress their immune status. The treatments were administered orally twice daily for 4 weeks. The positive control (RG treatment) is well known to improve immune status in experimental designs with several mouse types [31, 32]. The concentration of the probiotic culture media that we administered daily was calculated using FDA guidance [33].

### Systemic Immune Response Measurement

The animals were sacrificed by carbon dioxide inhalation and the spleens were quickly removed and cultured in plasticware. Single cell suspensions were prepared by mechanically homogenizing the spleens and passing them through a 70-μm cell strainer (Corning, USA). Splenocyte were extracted using RBC buffer (Sigma-Aldrich). After centrifugation and washing with PBS, the splenocytes were cultured in Roswell Park Memorial Institute 1640 medium (RPMI 1640; Hyclone, USA) supplemented with 10% fetal bovine serum, 100 U/ml penicillin, and 100 μg/ml streptomycin (Gibco) at 37°C in a 5% CO2 atmosphere. After final culturing of the splenocytes, total cell assays were subjected to ELISA analysis of systemic immune status using appropriate kits. Examples include INF-γ (SMIF00), IL-6 (SM6000B), IL-4 (SM4000B), and TNF-α (SMTA00B), all four from R&D Systems (USA). All ELISA kits were measured according to the manufacturer's instructions with no changes.

To regulate the immune status, we measured the activation of NK cells (G1780, Promega, USA) in the supernatant of the splenocyte culture media. The activity of NK cells as effector cells in spleen was evaluated using YAC-1 cells as a target cell (Korean Cell Line Bank). Splenocytes and YAC-1 cells [1 × 10^4^ cells/well] were cocultured to gain an effector-to-target cell ratio of 10:1. After 4 h of coculturing, the culture supernatants [50ul/well] were mixed with lactate dehydrogenase [LDH] solution (Pomega Corp., USA) and the absorbance value of each well was measured at 490 nm. The percentage of NK cellular cytotoxicity was calculated using the following formula: cytotoxicity [%] = [(experimental release - spontaneous release)/(maximum release – spontaneous release)] × 100.

### Fecal Microbiome Measurement

After 4 weeks, fecal samples from each mouse group were collected for gut microbiome measurement. This part of the experiment was shared with ChunLab, Inc. (Korea). First, feces collected from each treatment group were subjected to DNA extraction using a Fast DNA Spin Kit (MP Biomedicals, USA). The first PCR amplification was performed using a T100 thermal cycler (Bio-Rad) to amplify the V3 and V4 regions of 16S rRNA.

The amplifications were carried out under the following conditions: initial denaturation at 95°C for 3 min followed by 25 cycles of denaturation at 95°C for 30 s, primer annealing at 55°C for 30 s, and extension at 72°C for 30 s, with final elongation at 72°C for 5 min. The second PCR amplification was performed to attach the Illumina NexTera barcodes. Amplification was confirmed by 1% agarose gel electrophoresis and visualization of the PCR products using a Gel Doc system (BioRad). The PCR amplification products were purified using a QIAquick PCR Purification Kit (Qiagen, USA). Equal concentrations of purified products were pooled together and short fragments (non-target products) were removed with Ampure beads (Agencourt Bioscience, USA). The size and quality of the amplified products were assessed on a Bioanalyzer 2100 (Agilent) using a DNA 7500 chip. Mixed amplicons were pooled and sequencing was performed using the Illumina MiSeq sequencing system (Illumina). Analysis of the bioinformatics was quickly checked by ChunLab’s bioinformatics cloud platform. The linear discriminant analysis (LDA) effect size is presented in a bar plot and the parameters were set with default p-value, α = 0.05, and an LDA score of 2.0 with LEfSe.

### Statistical Analysis

Results were expressed as mean ± SEM. One-way analysis of variance (ANOVA) was used to assess the differences between groups. Post hoc analysis using Duncan’s multiple range test was performed, where significant effects were found, *p* < 0.05 was considered statistically significant.

## Results

### Concentration of Free Amino Acids in a Mixture of Two *Lactobacillus* and One *Bacillus* Strain

First, we investigated whether the probiotic cell-free supernatant composition involved metabolites. We studied three groups: aromatic amino acids, branched-chain amino acids (BCAA), and free amino acids (FAA). The aromatic amino acids included phenylalanine and tyrosine (480 and 194 mg/l), as well as tryptophan (not yet quantified). The BCAAs included valine, leucine, and isoleucine (526, 787, and 340 mg/l). The other metabolites found included seven amino acids: GABA, glutamine, cysteine, histidine, proline, lysine, and arginine (27, 766, 24, 102, 186, 484, and 325 mg/l). In these results, the aromatic amino acids and glutamine, lysine, and arginine were abundant in the 3-probiotic cell-free supernatants (Table 1). This means that the 3-probiotic cell-free supernatants contained an immune-stimulator compound [34-36].

### Postbiotics Mixture Enhanced TNF-α Expression in RAW264.7 Cells

**Fig. 1:**
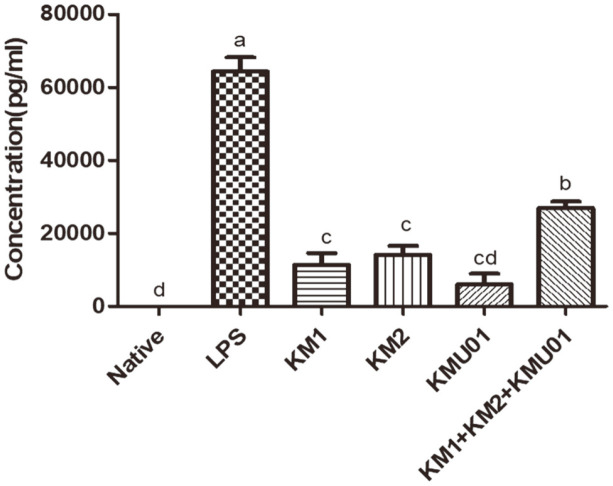
Postbiotics mixture enhanced the macrophage activation in RAW264.7 cells. After LPS (10 ng/ml) and each other postbiotics (*L. plantarum* KM1, *L. Plantarum* KM2, *B. velezensis* KMU01, each other postbiotics mixture=1:1:1, 1%) treatment, total cell sups were subjected to ELISA analysis. TNF-α concentration was measured using the manufacturer’s protocol. Means with different superscript letters are significantly different at *p* <0.05 as determined by Duncan’s multiple range test. Values are expressed as the mean ± S.E.M. (*n* = 3).

The use of postbiotics has recently been gaining attention because the administration of dead or inactivated cells reduces the risks associated with the administration of live bacteria, especially in immunocompromised individuals [37]. Moreover, the efficacy of postbiotics has been investigated to determine the protective effect of immune stimulation by lipopolysaccharide (LPS) on tissue damage [38, 39]. Thus, postbiotics mixtures were initially investigated to determine whether postbiotic treatment induced the expression of cytokine TNF-α in macrophages. The difference in expression of TNF-α was significant between the LPS and postbiotics treatment groups (Fig. 1). In addition, in the postbiotics mixture group there was significant decrease in the TNF-α expression levels compared with the LPS group. However, TNF-α expression was lower in the KM1, KM2, and KMU01 groups than in the postbiotics mixture group (Fig. 1).

### Increased NK Cell Activation Ameliorates Suppressed Immune Status Owing to the Postbiotics Administered

**Fig. 2:**
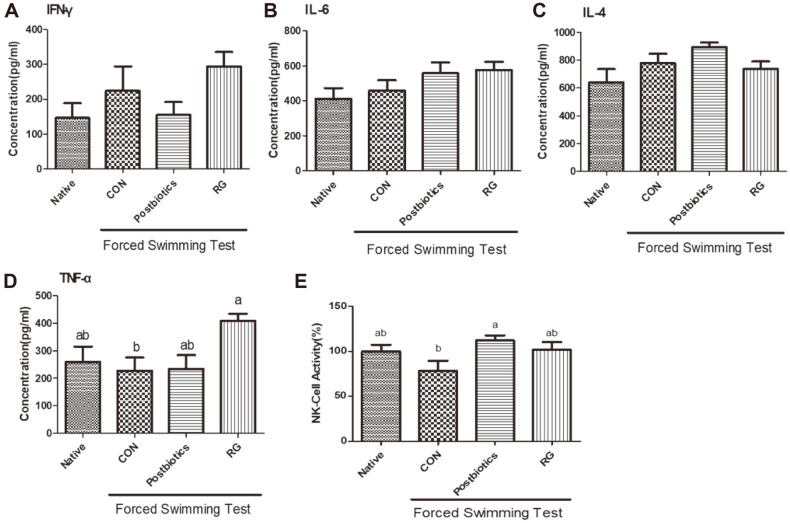
Immune-response cytokine expression accompanied by postbiotics mixture treatment in splenocyte. After postbiotics mixture and RG treatment, total splenocyte cell sups were subjected to ELISA analysis. Th1 (TNF-α, IFN-γ) and Th2 (IL-4, IL-6) concentration was measured using the manufacturer’s protocol. Means with different superscript letters are significantly different at *p* <0.05 as determined by Duncan’s multiple range test. Values are expressed as the mean ± S.E.M. Ten experimental animals were randomly assigned to each of 4 experimental groups: Native, CON, Postbiotics, and RG. Native group: mice received saline by daily gavage and were not forced to do the swimming test; CON group: mice received only saline by daily gavage and were forced to do the swimming test; Postbiotics group: mice received only saline by daily gavage of 400 μl of postbiotics mixture treatment; RG group: mice received only saline by daily gavage of 300 mg/kg of Red Ginseng (RG) treatment.

We investigated whether postbiotics mixtures via oral administration might mitigate suppressed immune status (Fig. 2). The expression of T-helper 1 (Th1), tumor necrosis factor-α (TNF-α), interferon-γ (IFN-γ), T-helper 2 (Th2), interleukin-4 (IL-4), and interleukin-6 (IL-6) was not shown to be significantly different between the Control group and the postbiotics mixtures treatment group (Figs. 2A-2D). However, the RG group had increased IFN-γ and IL-6 expression levels compared with the other experimental groups (Figs. 2A and 2B). In addition, the expression of TNF-α was significantly higher in the RG group than in the other experimental groups. The expression of IL-4 was highest in the postbiotics mixtures group, but did not show a statistically significant difference among the experimental groups (Fig. 2C). Moreover, NK cell activation was significantly different between the postbiotics mixtures treatment group and the CON treatment group (Fig. 2E). In the postbiotics mixtures group, NK cell activation showed a significantly higher concentration than with the other treatment groups.

### Composition of Gut Microbiome Influenced by Administration of Postbiotics Mixtures

**Fig. 3:**
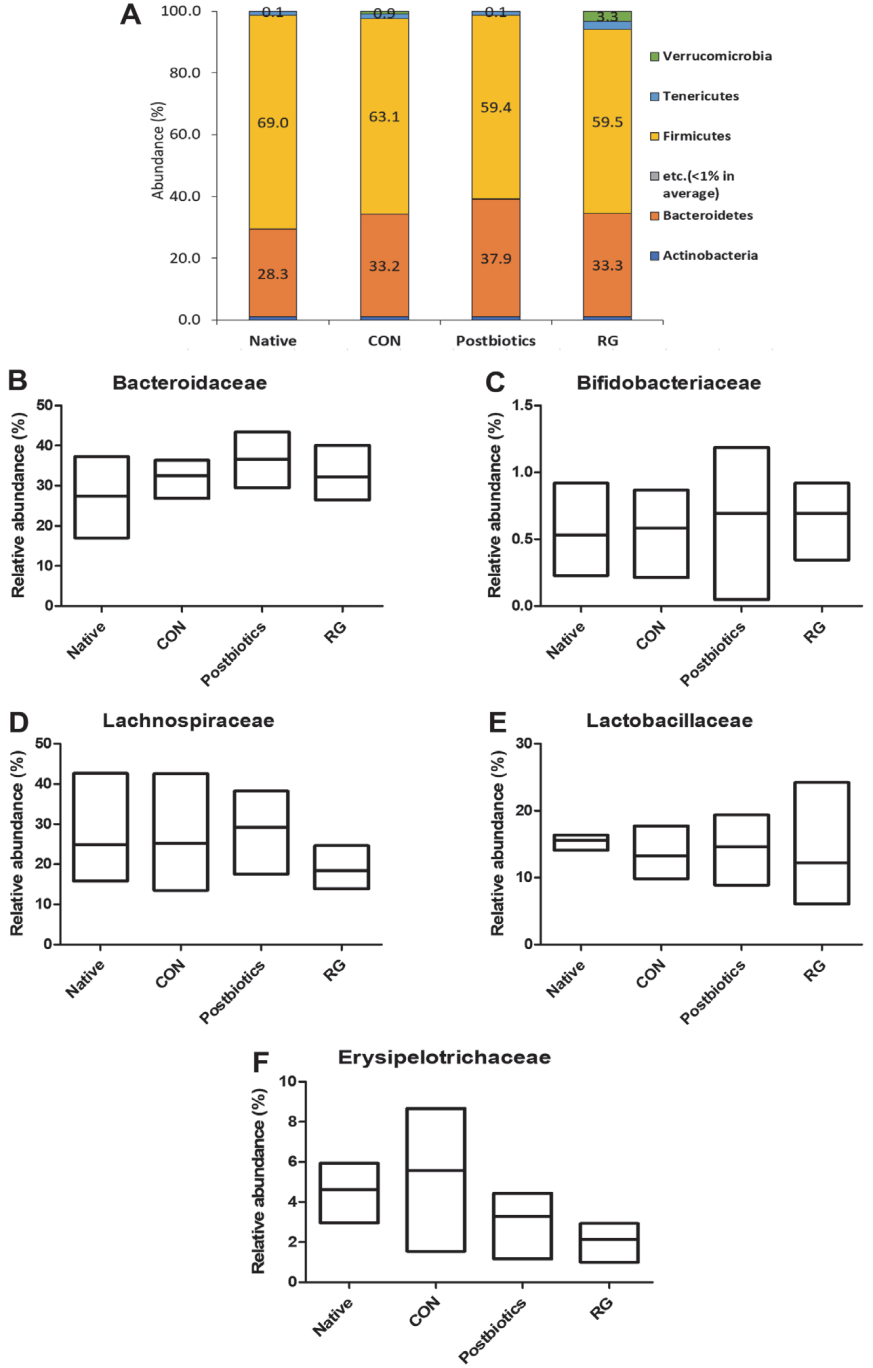
Compositional characteristics of gut microbiota at the phylum and family level. The relative abundance of all experimental groups on the gut microbiota at the phylum level (**A**). The relative abundance of *Bacteroidaceae* (**B**), *Bifidobacteriaceae* (**C**), *Lachnospiraceae* (**D**), *Lactobacillaceae* (**E**), and *Erysipelotrichaceae* (**F**) in the different groups at the family level. Native group: mice received saline by daily gavage and were not forced to do the swimming test; CON group: mice received only saline by daily gavage and were forced to do the swimming test; Postbiotics group: mice received only saline by daily gavage of 400 μl of postbiotics mixture treatment; RG group: mice received only saline by daily gavage of 300 mg/kg of Red Ginseng (RG) treatment.

To determine the impact of postbiotics mixtures on the mouse gut microbiota, 16S sequencing of bacterial rRNA was performed on the fecal content. Firmicutes was the predominant phylum in the feces of all experimental groups. The proportion of Bacteroidetes was slightly higher in the postbiotics mixtures group than in other experimental groups, although without statistical difference (Fig. 3A). At the family level, *Bacteroidaceae* (Fig. 3B), *Bifidobacteriaceae* (Fig. 3C), and *Lachnospiraceae* (Fig. 3D) were the highest in the postbiotics mixtures group. The proportion of *Lactobacillaceae* (Fig. 3E) was higher than in the CON and RG groups, but the difference was not statistically significant. In the CON group, the abundance of *Erysipelotrichaceae* was greater than in the other experimental groups, although without statistical difference (Fig. 3F).

**Fig. 4:**
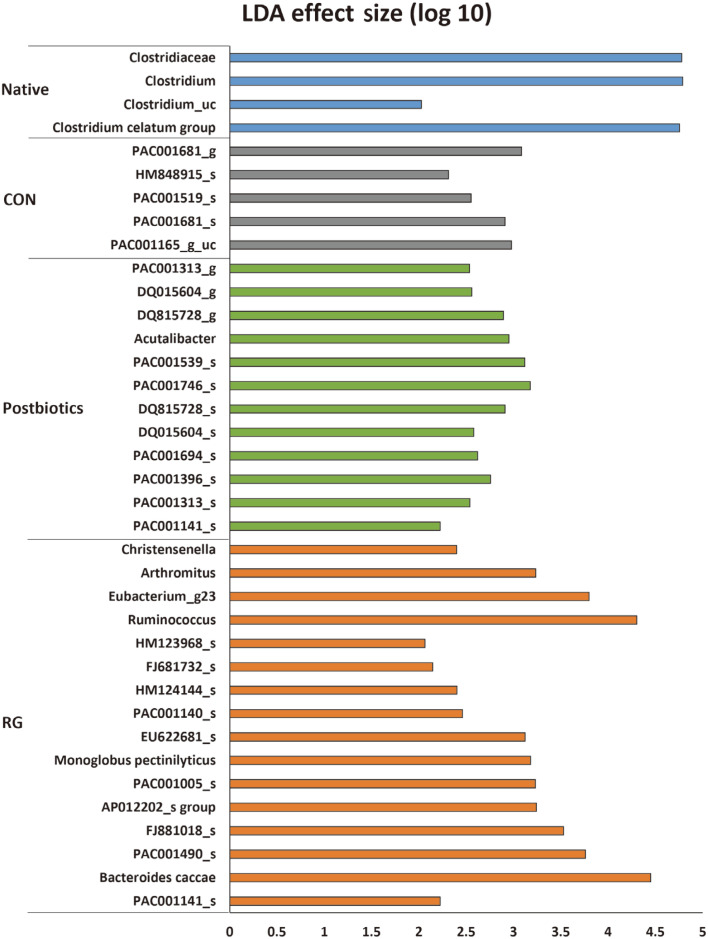
The linear discriminant analysis effect size (LEfSe) analysis of microbial abundance among different fecal samples. Linear discriminant analysis effect size (LEfSe) analysis results comparing all four groups. Significance obtained by LDA effect size at *p* < 0.05, (Kruskal-Walis test) and LDA score > 2.0. Native group: mice received saline by daily gavage and were not forced to do the swimming test; CON group: mice received only saline by daily gavage and were forced to do the swimming test; Postbiotics groups: mice received only saline by daily gavage of 400 μl of postbiotics mixture treatment; RG group: mice received only saline by daily gavage of 300 mg/kg of Red Ginseng (RG) treatment.

In the LEfSe analysis (Fig. 4), in the Native group, the abundance of *Clostridiaceae*, *Clostridium*, *Clostridium_uc*, and *Clostridium celatum* was higher than in the other experimental groups. The abundance of *PAC001681_g*, *HM848915_s*, *PAC001519-s*, *PAC001681_s*, and *PAC001165_g_uc* was highest in the CON group compared to the other experimental groups.

In the postbiotics mixtures group, the abundance of *PAC001313_g, DQ015604_g, DQ815728_g, Acutalibacter, PAC001539_s, PAC001746_s, DQ815728_s, DQ015604_s, PAC001694_s, PAC001396_s, PAC001313_s*, and *PAC001141_s* was higher than in the other experimental groups (Fig. 4).

In the RG group, the abundance of *Christensenella*, *Arthromitus*, *Eubacterium_g_23, Ruminococcus, HM123968_s, FJ681732_s, HM124144_s, PAC001140_s, EU622681_s, Monoglobus pectinilyticus, PAC001005_s, AP012202_s group, FJ881018_s, PAC001490_s, Bacteroides caccae*, and *PAC001141_s* was higher than in the other experimental groups (Fig. 4).

## Discussion

In the present study, we tested the effect of postbiotics on the immune status imbalance induced by suppressing the immune status of mice subjected to a forced swimming test. Although the relationships between postbiotics and immune status has not been fully elucidated, it is well accepted that probiotics enhance the immune status and inflammatory mechanism. This is because probiotics often induce the enhancement of immune, gut health, and anti-inflammatory response pathways under stress conditions [5-12].

The definition of postbiotics was assigned after the International Scientific Association for Probiotics and Prebiotics (ISAPP) made a presentation online in 2019. Postbiotics were defined as “A preparation of inanimate microorganisms and/or their components that confer a health benefit on the host.” Postbiotics are primarily associated with immunomodulatory activities, because they play a role in boosting the innate and adaptive immune system, maintain the integrity of the intestinal mucosal barrier and antagonize pathogens using antimicrobial compounds, similar to the effects of probiotics [14].

Herein, we demonstrated the enhancement of NK cell activation after the administration of postbiotics mixtures from suppressed immune status induced by a forced swimming test. Treatment with the postbiotics mixture stimulated macrophage cell activation. This means that cell-contact-dependent activation is related to macrophage-NK cell activation. Previous studies have shown that NK cell surfaces respond to the stimulated macrophage cell, depending on the pathogen [40]. Our finding indicates that postbiotics regulate macrophage activation after administration of postbiotics mixtures.

Postbiotics improve the status of a suppressed immune condition, and are proven to be effective on Th1 and Th2-linked cytokine expression. Th1-linked cytokine, IFN-γ, plays an important role in modulating the immune response (including host defense against intracellular pathogens). As well as NK cells, IFN-γ produces NK T cells, CD8^+^ T cells, and T-helper 1 (Th1) CD4^+^ T cells [41].

Recently, it has been shown that cell metabolism regulates the IFN-γ expression of NK and T cells [42-47] and specific metabolic requirements of NK-cell IFN-γ production in mice and humans [48].

However, postbiotic treatment also decreases the expression of IFN-γ compared with the control group, while its expression is enormous in the RG treatment group. Furthermore, IFN-γ inhibits immune response Th2, while IL-4 inhibits immune response Th1 [41, 49-52]. Previous studies have shown that IFN-γ production of murine NK cells cultured in the presence of IL-4 has not been detected [53]. Exposure to IL-4 also suppressed the cytotoxic ability of NK cells with in vitro and in vivo models [54, 55]. IFN-γ and IL-4 display opposing regulatory roles in the immune system. It is interesting to note that there are numerous seemingly contradictory reports on the effect of IL-4 on NK cells, and the role of IL-4 for NK cells is still unclear. According to these findings, we also found that IL-4 expression was increased in the postbiotics mixture treatment group, while IFN-γ expression was decreased. In addition, IL-6 stimulates Th2. Therefore, the Th2-mediated response is B-cell activity that is involved in innate immunity activation. As a result, it can be speculated that the increased IL-6 is involved in immune activation and regeneration [56]. It has also been reported that expression of TNF-α is correlated with the level of IL-6 expression [57].

Several probiotic strains have been studied to examine their beneficial effect on NK cell activity. Strains of *Bacillus polyfermenticus*, commonly called bispan strains, are used to treat certain conditions of the intestinal wall [6, 58]. A bispan strain increased the levels of NK cell activation, which was proven in an eight-week clinical test [59]. In accordance with previous study, we also examined NK cell activation using one of the three types of probiotics (*Bacillus velezensis* strain). The latter strain has been identified with 16s rRNA sequences from *Bacillus polyfermenticus*, so its functional efficacy is the same as that of *Bacillus polyfermenticus* [60]. Nevertheless, the metabolites of probiotics, that is to say, their actions directly on the activation of NK cells, are unclear.

Under elevated NK cell activation, postbiotics regulate the immune system and modulate the gut microbiome. The treatment with postbiotics mixtures was able to modulate abundance at the family level, including Bifidobacteria, *Lachnospiraceae*, and *Lactobacillaceae*. In general, *Lactobacillus* strains spp. and *Bifidobacterium* strains spp. produce the immunostimulant proteins associated with bacteria cell walls, such as lipoteichoic acid and peptidoglycan, as postbiotics. These composite activities have been reported to maintain equilibrium between cytokine concentrations associated with Th1 and cytokine concentrations associated with Th2 [61, 62]. In addition, anti-inflammatory cytokine and pro-inflammatory cytokine expressions have been mediated as well [63]. Moreover, previous studies have reported that *Lachnospiraceae* is a family of anaerobic bacteria that are important short-chain fatty acid (SCFA) producers that reside among the gut microbiota [64]. SCFAs are important metabolites for maintaining gut homeostasis. Several studies have shown that SCFAs, and in particular butyrate, also have important immunomodulatory functions [65]. In the CON group, the abundance of *Erysipelorichaceae* exhibited a trend of increase compared to the other experimental groups. The *Erysipelotrichaceae* family are known as opportunistic pathogens [66]. According to several studies, our postbiotics show immunomodulatory activity and that it is beneficial for the health of the host.

In this study, we show that postbiotics mixtures lead to enhancement of NK cell activation, accompanied by increase in the quantity of obligate anaerobe bacteria. Enhancement of NK cell activity results in increased immune-related microbiome modulation in mice in which stress was induced using a forced swimming test. Induction of obligate anaerobe bacteria may therefore alter the gut total microbiome, leading to immune system activation in the form of Th1/Th2 expression related to NK cell activation. These findings suggest that postbiotics mixtures may stimulate NK cell activation through gut microbiome-immune system interactions.

## Figures and Tables

**Table 1:** Concentration of free amino acids in postbiotics mixture.

Free amino acids	Concentration (mg/l)
Phenylalanine	480
Tyrosine	194
Tryptophan	
Valine	526
Leucine	787
Isoleucine	340
GABA	27
Glutamine	766
Cystine	24
Histidine	102
Proline	186
Lysine	484
Arginine	325
Total	4,241
